# Microsatellite-based genetic diversity and population structure of domestic sheep in northern Eurasia

**DOI:** 10.1186/1471-2156-11-76

**Published:** 2010-08-10

**Authors:** Miika Tapio, Mikhail Ozerov, Ilma Tapio, Miguel A Toro, Nurbiy Marzanov, Mirjana Ćinkulov, Galina Goncharenko, Tatyana Kiselyova, Maziek Murawski, Juha Kantanen

**Affiliations:** 1 Biotechnology and Food Research, MTT Agrifood Research Finland, 31600 Jokioinen, Finland; 2 Departamento de Produccion Animal, Universidad Politecnica de Madrid, 28040 Madrid, Spain; 3 All-Russian Research Institute of Animal Husbandry, Russian Academy of Agricultural Sciences, 142132 Moscow Region, Dubrovitsy, Russia; 4 Animal Science Department, University of Novi Sad, 2100 Novi Sad, Serbia; 5 Siberian Branch of Russian Academy of Agricultural Science, 630501 Novosibirsk Region, Krasnoobsk, Russia; 6 All-Russian Research Institute of Animal Genetics and Breeding, Russian Academy of Agricultural Sciences, 189620 St. Petersburg-Pushkin, Russia; 7 Department of Sheep and Goat Breeding, Agricultural University of Cracow, 31059 Cracow, Poland

## Abstract

**Background:**

Identification of global livestock diversity hotspots and their importance in diversity maintenance is essential for making global conservation efforts. We screened 52 sheep breeds from the Eurasian subcontinent with 20 microsatellite markers. By estimating and weighting differently within- and between-breed genetic variation our aims were to identify genetic diversity hotspots and prioritize the importance of each breed for conservation, respectively. In addition we estimated how important within-species diversity hotspots are in livestock conservation.

**Results:**

Bayesian clustering analysis revealed three genetic clusters, termed Nordic, Composite and Fat-tailed. Southern breeds from close to the region of sheep domestication were more variable, but less genetically differentiated compared with more northern populations. Decreasing weight for within-breed diversity component led to very high representation of genetic clusters or regions containing more diverged breeds, but did not increase phenotypic diversity among the high ranked breeds. Sampling populations throughout 14 regional groups was suggested for maximized total genetic diversity.

**Conclusions:**

During initial steps of establishing a livestock conservation program populations from the diversity hot-spot area are the most important ones, but for the full design our results suggested that approximately equal population presentation across environments should be considered. Even in this case, higher per population emphasis in areas of high diversity is appropriate. The analysis was based on neutral data, but we have no reason to think the general trend is limited to this type of data. However, a comprehensive valuation of populations should balance production systems, phenotypic traits and available genetic information, and include consideration of probability of success.

## Background

The domestic sheep (*Ovis aries*) has been an economically and culturally important farm animal species since its domestication in the Near East approximately 9,000 years B.P. [1]. A northern Eurasian sheep stock formed some 6,000 years ago as sheep were brought to the British Isles, northern Europe and Russia after the expansion to the European continent via Danubian and Mediterranean routes [2], and a possible route through Russia [3]. Sheep dispersed across Europe in temporally separate migratory episodes: the most original and a more primitive type of domestic sheep was later replaced by a more developed wool type of sheep. Ancestry from the first immigrant wave seems to have survived only in north-western and northern peripheries of Europe [4].

A similar replacement process is occurring in modern days. Global standardization of production environments and breed competition have led to the disappearance of many native breeds. Food and Agriculture Organization of the United Nations (FAO) has estimated that 36% of the sheep breeds of known census size are either extinct or endangered [5]. Furthermore, the use of a few high-quality males for intense mating has resulted in the reduction of effective population size (N_e_) over time and reduced genetic diversity within breeds [6]. These processes will lead to the decrease of effective population size of the entire species. This could restrict breeding options and genetic gain of breeding programs to the extent that unpredictable future requirements might not be met [6-8]. Breed conservation aims to maintain these options, but limited resources, e.g. financial limitation, might not allow conservation of all the breeds.

One can argue that the breeds originating from or close to the domestication centers, such as the Near Eastern region, should be particularly prioritized in conservation programs. Microsatellite studies in cattle (*Bos taurus*) [9-11], goat (*Capra hircus*) [12] and sheep (*Ovis aries*) [13] suggested that the breeds located close to the putative domestication centers are the most variable. These breeds might possess allelic variations retained from the wild ancestors that never reached areas further from the center of origin. Although one cannot easily differentiate these primary diversity hotspots from the secondary hotspots created by a more recent crossbreeding, continent-wide mapping of the regions of exceptional livestock diversity (genetic diversity hotspots) has been suggested as a means of targeting conservation efforts for livestock species [10,14]. DNA marker data can be used to calculate molecular coancestries within and between breeds and determine contributions of each breed to a pool of animals that would maximize genetic diversity of the pool, i.e. minimize average molecular coancestry [15]. These calculations can provide critical information when the prioritization of breeds needs to be done for conservation of diversity of domestic animal species. Using this conservation approach, it would be possible to maximize N_e _of the subdivided species and thus minimize the depleting effect of genetic drift on genetic variation.

There have been a few quite comprehensive gene diversity studies in sheep [13,16-18]. However, none of these focused on breed prioritization to describe general trends in the conservation of genetic diversity in sheep. Though genome-wide Single Nucleotide Polymorphisms (SNP) data are becoming the standard for livestock genetics [18], they can have a problem of ascertainment bias originating from SNP discovery protocols [19]. Though the problem can be alleviated through using haplotypic measures [20] or through bias corrections [21], the established baseline trend using low bias markers such as microsatellites remains an important benchmark. We used a representative set of sheep types across the Northern Eurasia to explore the diversity patterns and inferred conservation priorities based on microsatellites. For breed ranking we applied the method based on the minimization of molecular coancestry in a subdivided population by Caballero and Toro [15]. We tested the effect by weighting differently the two components of maximum genetic diversity, within-breed and between-breed variation, when doing priority settings of breeds. Based on the common statement that populations from diversity hotspot regions are more important [9,14,10], we expected large number of breeds from a hotspot region to be highly prioritized.

## Results

### Genetic diversity

In total, 342 alleles were detected at the 20 microsatellite loci analyzed (Additional file 1: Table S1). A summary of the genetic diversity parameters computed for 16 regional groups is presented in Table 1 and the breed-wise values based, on an average, on 32 sheep per breed are given in Additional file 2: Table S2. The total genetic diversity (H_T _) varied from 0.651 to 0.807 in the Danish and the Ukrainian regional groups, respectively. The area having regional groups with H_T _values above 0.8 (Ukraine, southeast Europe, Kazakhstan and east of the Caspian Sea, Buryatia and the southern Caucasus), was termed a diversity hotspot. Among breeds, the unbiased expected heterozygosity (H_S_) ranged from 0.613 (the Norwegian Cheviot) to 0.806 (the Russian Karakul), with an average value of 0.759. Allelic richness varied in the similar pattern as other within-population diversity measures (e.g. *H*_T _and *H*_S _) across the breeds (Table 1). The overall estimate of *f *[22] was 0.011. The breed-wise *f *estimates were significantly (*P *< 0.05) greater than zero only for the Norwegian Rygja Sheep and the Swedish Rya Sheep suggesting that most breeds are quite uniform (Additional file 2: Table S2).

**Table 1:** Genetic diversity within 16 regional groups

Geographical region	Regional group	N	**H**_**T**_	*f*	R
Caucasus	South Caucasus*	6	0.802	0.020	6.57
	North Caucasus	5	0.795	0.017	6.24
	Stavropol	3	0.792	-0.005	6.04
	Caspian depression	3	0.795	0.023	6.19
Asia	Kazakhstan and east of Caspian Sea*	6	0.804	0.037	6.45
	Altai	2	0.795	0.004	6.41
	Buryatia*	2	0.802	-0.008	6.42
Eastern fringe of Europe	Volga region	2	0.779	0.015	5.88
	West Russia	3	0.794	-0.041	5.45
	Ukraine*	2	0.807	-0.004	6.43
	Southeast Europe*	4	0.806	-0.006	6.14
	Poland	3	0.759	-0.015	6.24
	Finland	2	0.758	0.007	5.35
	Scandinavia	6	0.774	0.030	4.59
	Denmark	1	0.651	0.028	4.41
	Iceland and Faeroe Islands	2	0.746	-0.001	4.95

Number of breeds (N), total gene diversity (H_T_), departures from Hardy-Weinberg equilibrium (*f*), and mean allelic richness (R).

* Regional groups identified as diversity hotspots.

### Genetic cluster analysis

**Figure 1:**
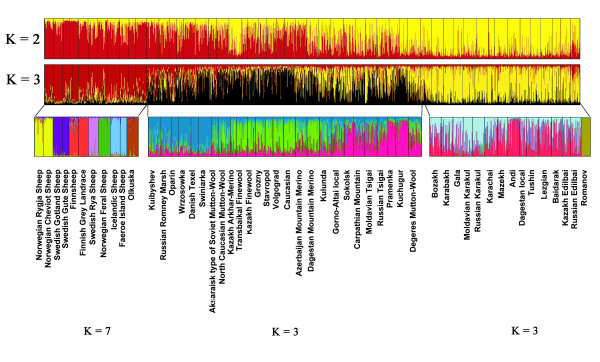
**Clustering of 52 sheep breeds**. Individuals are presented as vertical lines divided into *K *colors, representing constructed populations. The lowest row represents further clustering of 3 groups, identified at *K *= 3, separately. The Nordic group is divided into 7 subclusters, while the Composite (in the middle) and the Fat-tailed groups each split into 3 subclusters.

A model-based clustering was applied to resolve the population genetic structure. At *K *= 3, one cluster was constituted by the breeds descending mainly from the northernmost edge of the studied distribution (termed the 'Nordic cluster'), while the fat-tailed breeds, originating mainly from the Caucasus and Caspian basin areas, geographically close to the Near Eastern domestication center, formed the second cluster (termed the 'Fat-tailed cluster'). A third cluster mainly contained the composite sheep breeds from central Eurasia (termed the 'Composite cluster') (Figure 1). The mean similarity coefficient (SC) across 10 runs was 0.984 at *K *= 3. At *K *= 4, a split within the Nordic cluster was observed, but the drop of SC to 0.534 indicated variable assignments for breeds across runs and lack of additional strong high-level substructure among the populations. Therefore, separating the entire dataset into three clusters was chosen as the final global configuration.

To dissect the genetic structure within the three clusters, STRUCTURE analysis was further applied to each of them separately. In the Nordic cluster, the most consistent grouping of 11 north European sheep breeds was achieved at *K *= 7 (SC = 0.641), with the mean SC ranging from 0.250 to 0.314 at *K *other than seven. Breeds originating from the same country (e.g. Finnsheep and Finnish Grey Landrace) or from the neighboring regions (e.g. the Icelandic Sheep and the Faeroe Island Sheep) tended to cluster together (Figure 1).

The Fat-tailed sheep cluster was composed mainly of the coarse-wool native breeds from the Caucasus and steppes of the Caspian basin and Kazakhstan. Surprisingly, the northern short-tailed coarse-wool Romanov Sheep was also assigned into this cluster. The breed's estimated fraction of the Fat-tailed cluster was 0.59. However, the most consistent subclustering of the Fat-tailed cluster was obtained at *K *= 3 (SC = 0.865), with the Romanov sheep forming a distinct subcluster (Figure 1). The Andi and the Karakul type sheep breeds anchored the remaining two subclusters. Eight out of 14 breeds showed partial and varying memberships of the two subclusters, indicating their admixed origin (Figure 1).

The Composite cluster hosted the remaining 26 synthetic semi- and fine-wool sheep breeds that were split into three genetic subclusters (SC = 0.932). The three subclusters identified followed a pattern of geographical separation: long-wool Marsh and Texel type breeds from the north grouped into subcluster I (light blue); fine-wool breeds from the Caucasus, Kazakhstan and Buryatia formed subcluster II (light green) and the southern European Zackel type breeds grouped into subcluster III (pink, Figure 1). Other nine European, Caucasian and Asian sheep breeds had partial membership of multiple clusters, which represents more diverse ancestries in the process of breed development (Figure 1).

**Figure 2:**
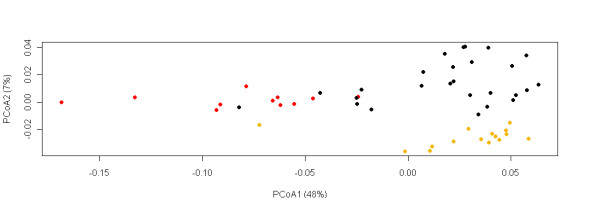
**Principal coordinate plot of breeds based on Chord distance**. Axis I explains 48% of the variation, axis II explains 7% of the variation. Breeds from Nordic cluster (based on STRUCTURE) are marked with red, breeds from Fat-tailed cluster are marker with yellow and breeds from the Composite cluster are marked with black circles.

The PCoA results were quite in accordance with the STRUCTURE results. The breeds from the above mentioned Nordic cluster were separated from the other breeds on Axis I, which explained 48% of the distance matrix (Figure 2). On Axis II, breeds from the Fat-tailed cluster were separated from the Composite cluster breeds, which explained an additional 7%. A notable exception was the Romanov Sheep whose yellow circle in Figure 2 (at -0.085,-0.004) suggested the breed's clustering with the Nordic rather than the Fat-tailed breeds (Figure 2). This matched our prior expectations on the basis of phenotypic characters better than the STUCTURE result.

**Figure 3:**
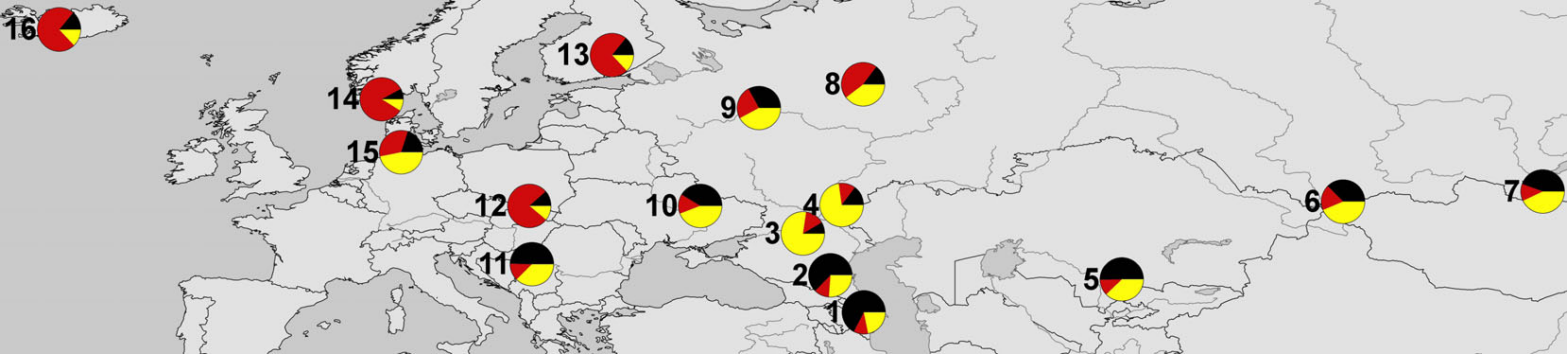
**Distribution of three inferred genetic clusters in the study regions**. Slices in the pie diagrams represent Fat-tailed (yellow), Composite (black) and Nordic (red) clusters. The Caucasus area is represented by four regions: South Caucasus (1), North Caucasus (2), Stavropol (3) and the Caspian depression (4). The Asian region is represented by three regions: Kazakhstan and east of the Caspian Sea region (5), the Altai region (6) and the Buryatia region (7). The remaining groups belong to eastern fringe of Europe: the Volga region (8), West Russia (9), Ukraine (10), Southeast Europe (11) Poland (12), Finland (13), Scandinavia (14), Denmark (15) and Iceland and the Faeroe Islands (16).

The proportions of Nordic, Fat-tailed and Composite genetic ancestries within each of the regional groups studied are presented in Figure 3. The highest proportion of Fat-tailed ancestry was recorded at the southern periphery of the studied distribution, which gradually decreased northwards and was the smallest in the northern regional groups. The proportion of Nordic type ancestry mirrored this pattern and was the largest in the northern regional groups and decreased southwards. The 16 regional groups had similar proportions of Composite ancestry, with the exception of Stavropol and Caspian depression regional groups, where the proportion of Composite ancestry was highest, and the northernmost and southernmost regional groups, where the Composite ancestry proportion was least.

### Geographical patterns in genetic diversity

**Figure 4:**
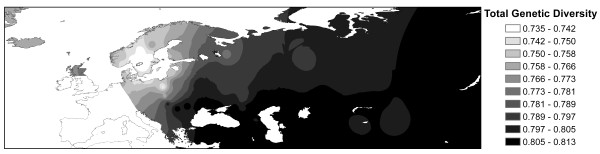
**Contour synthetic map of total genetic diversity (*H***_**T**_**) calculated for triplets of neighboring breeds**. Darker shading indicates higher levels of diversity.

To reduce the effect of possible recent breed-specific factors on the overall geographical distribution of genetic diversity, a synthetic map of genetic diversity was based on the total gene diversity (*H*_*T*_) for triplets of neighboring breeds (Figure 4). The highest diversity was found in the southern region of the studied area: Buryatia (south Siberia), Caspian Sea and Black Sea basins. It decreased gradually in Central and northern Europe and the lowest *H*_*T *_values were recorded for southern Scandinavia (Figure 4). The trend can be observed also based on within breed estimates (Additional file 3: Figure S1). A significant but weak positive correlation (*r *= 0.382, *P *< 0.05) was calculated between the expected heterozygosity and the level of admixture based on global STRUCTURE results (*K *= 3) for the 52 sheep breeds studied, suggesting that admixture does not explain the presence of diversity hotspots, though it can contribute to it in some areas.

### Analysis of molecular variance (AMOVA)

We tested the extent of population differentiation using AMOVA in the whole dataset, as well as grouping breeds according to geographical regions (the Caucasus, Asia, or the eastern fringe of Europe), and according to the 15 regional groups (excluding the Danish group represented by a single breed) (Table 2). As expected, most genetic variation (> 86%) was retained within the breeds, whereas only 0.41% to 0.95% (*P *< 0.001) of the variation could be explained by geographical partitioning (Table 2). The between-breed variation within each of the three genetic subclusters was significant (*P *< 0.001), ranging from 2.48% (Fat-tailed subcluster) to 13.71% (Nordic subcluster) (Table 2). In our data, using genetic clustering in AMOVA gives higher between groups variance than using geographical categorizations.

**Table 2:** Analysis of molecular variance

Sample	Number of breeds	Number of breed groups	Percentage of variance and significance (*P*)		
			
			Within breeds	Among breeds within groups	Among groups
Whole data	52	1	93.56 (< 0.001)	6.44 (< 0.001)	
Three geographical regions*	52	3	93.43 (< 0.05)	6.17 (< 0.001)	0.41 (< 0.001)
15 regional groups*	51	15	93.68 (< 0.001)	5.38 (< 0.001)	0.95 (< 0.001)
Three structure clusters:	52	3	93.13 (< 0.001)	5.63 (< 0.001)	1.24 (< 0.001)
Nordic subcluster	11	1	86.29 (< 0.001)	13.71 (< 0.001)	
Composite subcluster	26	1	95.45 (< 0.001)	4.55 (< 0.001)	
Fat-tailed subcluster	15	1	97.52 (< 0.001)	2.48 (< 0.001)	

* See Table 1 and supporting information for details.

### Core-set analysis

Of the 52 sheep breeds 24 had contributions to the core-set when the 4 weightings (λ = 0, 0.2, 0.5, 1) of within-breed diversity were considered. The 24 breeds represented all the 16 regional groups, except those of the Altai and Buryatia regions. The distribution of breeds was relatively even, with 1 to 2 sheep breeds per region, the exception being the Scandinavian regional group, which contributed 5 sheep breeds to this accumulated core-set (Additional file 4: Table S3). Looking at the four core-sets separately, the number of contributing breeds increased from 8 to 17 when weight of within-breed variability increased from 0 to 1 (Table 3). Results of analysis based on genetic clustering are comparable to those based on geographic regions (Additional file 5: Table S4).

**Table 3:** Distribution of core-set contributions

Geographical	Regional group	λ = 0		λ = 0.2		λ = 0.5		λ = 1	
		
region		Breeds	Cont	Breeds	Cont	Breeds	Cont	Breeds	Cont
Caucasus	South Caucasus*	0	0	0	0	0	0	2	0.17
	North Caucasus	0	0	0	0	1	0.04	1	0.01
	Stavropol	0	0	0	0	0	0	2	0.04
	Caspian depression	0	0	0	0	0	0	1	0
Asia	Kazakhstan and* east of Caspian Sea	0	0	0	0	0	0	1	0.14
	Altai	0	0	0	0	0	0	0	0
	Buryatia*	0	0	0	0	0	0	0	0
Eastern fringe	Volga region	0	0	0	0	0	0	1	0.06
of Europe	West Russia	1	0.04	2	0.11	2	0.20	1	0.03
	Ukraine*	0	0	0	0	0	0	2	0.24
	Southeast Europe*	0	0	0	0	1	0.04	1	0.10
	Poland	1	0.11	1	0.09	1	0.05	1	0.02
	Finland	0	0	0	0	0	0	1	0.03
	Scandinavia	4	0.56	4	0.51	5	0.42	2	0.10
	Denmark	1	0.19	1	0.17	1	0.13	0	0
	Iceland and Faeroe Islands	1	0.10	1	0.11	2	0.13	1	0.06

Sum		8	1	9	1	13	1	17	1
SD		1.03	0.14	1.09	0.13	1.33	0.11	0.68	0.07

Number of breeds (Breeds) and the sum of their optimal contributions (Cont) to the core set for each regional group using four different weightings (λ) for the within-breed variation.

* Regional groups in diversity hotspot.

Every tested scenario with reduced weight for within-breed variation (λ < 1) gave a significantly higher number of breeds with non-zero contribution from the areas *outside *the hotspot regions (all the two-tailed *P *values < 0.02 using Fisher's exact test for independence; Table 3, Additional file 4: Table S3). Looking at the contribution to the core-set, these non-hotspot region populations comprised of > 90% of the set. However, optimizing for global diversity (λ = 1), there is no significant difference (the two tailed *P *= 0.77) in the proportion of breeds included between hotspot and non-hotspot regions. Very distinctively, now the core-set consists of 65% of the breeds from the hotspot regions because the included hotspot region breeds make a significantly larger mean contribution (each ~11%) than the included breeds from the non-hotspot regions (each ~3%) (Welch two sample t-test *P *= 0.009). Thus the diversity hotspot areas were important for conserving total genetic diversity in terms of the effort per conserved population rather than proportion or number of breeds to be conserved.

Conservation programs might be initiated with limited information. In cases where resources allow keeping only a small number of breeds and when there is no aim to differentiate between their contributions to the core-set (assuming equal contributions), the maximum amount of genetic diversity would be maintained by giving priority to breeds from the diversity hotspot regions. In the scenario of 5 breeds, four of them are from the hotspot areas (Additional file 6: Table S5). The proportion of hotspot breeds was reduced from 80% to 45% when assuming resources to keep 20 breeds. This latter set is similar to 17 breeds identified as contributors to the core-set when λ = 1 (Additional file 4: Table S3), but includes also three fat-tailed populations from the Caucasus (Bozakh, Tushin and Lezian). These results agree with the idea of having the initial conservation focus on hotspot regions.

## Discussion

We present here a comprehensive genetic analysis of sheep populations originating from a broad geographical area of the Eurasian subcontinent. Our results detected the presence of a sheep genetic diversity hotspot located close to the Near East, the assumed sheep domestication center, and highlight the importance of such an area in conservation planning. The results correspond well with the geographical pattern of genetic diversity distribution reported for cattle (*B. taurus*) [23] and goat (*Capra hircus*) [12] as well as a previous study of European sheep [13] which focused on more southern breeds. The congruence across studies suggests the pattern to be genuine, though larger number of markers could be desirable. Based on observed allele number, we can expect the reliability to be approximately similar as in a study of 300-400 unbiased bi-allelic SNPs [24]. However, since studies of humans do not suggest great discrepancies across nuclear marker types as long as ascertainment bias can be avoided [19,20,25], we expect the presented general diversity patterns to be robust. Since in our analysis conservation optimization was based on the same data used to define the diversity hotspot, our general recommendation for considering hotspot regions ought to be sound.

Livestock genetic diversity hotspots have been suggested to be very important for conservation because the domestic animal stocks associated with them might possess allelic variation from wild ancestors, which, due to a sequence of founding events, was lost during the dispersion of animals towards the northern parts of the continent [14]. However, to the best of our knowledge this question has not been directly addressed previously. Our results provide additional evidence for the importance of these regions, while indicating an important refinement for the conservation goal. Our results do not suggest that a larger proportion of populations from these areas needs to be conserved, but rather suggest more emphasis be placed on each conserved diversity hotspot population. This distinction, however, is highly relevant for domestic species, where management units are in most cases clearly definable as breeds. Further the results support for directing the first conservation resources to work on hotspot regions.

Of the three identified Northern Eurasian genetic clusters, the Nordic cluster was represented by native and old commercial sheep breeds adapted to live under cold and wet northern European climatic conditions. This group includes breeds such as Gute, Icelandic Sheep and Finnsheep, which descended from the sheep stock in the first dispersion event to Europe [4]. Strict breed boundaries over a long period and geographical isolation, particularly for insular breeds (the Icelandic Sheep and the Faeroe Island Sheep), are characteristic of the group and have resulted in a unique and genetically highly heterogeneous pool of Nordic sheep populations (Table 2).

The large Composite cluster with partial ancestry from improved western breeds contains genetically variable fine- and semi-fine-wool sheep breeds of admixed origin with moderate differentiation between the breeds. The presence of substructure within the cluster reflects the differences in the breeding trends within the former Soviet Union that took place in the middle of the last century. The sheep in the western part of Russia and Volga regions have Marsh-Texel type composite ancestry resulting from crossing local populations with British type long-wool sheep (Figure 1). The second subcluster within the Composite group includes the breeds prevalent in the Caucasus, the Stavropol region and the Caspian basin, another geographical center of purposeful crossbreeding, with a significant genetic component of the Merino type sheep. The third subcluster within the Composite cluster is anchored by two Zackel type mountain sheep populations, Pramenka and Kuchugur, and reflects a common ancestry for the majority of breeds within the subcluster. The grouping of Tsigai in the same subcluster confirms the assumption that this breed was strongly influenced by Zackel (e.g. see [17]). Most of the populations of the Composite cluster also represent genetic diversity of local origins as the upgrading was performed on the basis of local sheep populations, mixing them with a number of improved breeds of foreign ancestry to combine desired production and robustness characteristics.

The Fat-tailed cluster hosted very variable native coarse-wool populations, living under a variety of climatic conditions, ranging from semi-desert and steppe regions around the Caspian Sea and Central Asia to Caucasian mountain terrains. The differentiation of fat-tailed sheep from the others indicates restricted gene flow between steppe or mountain environments in central Eurasia and cooler and moister northern areas of the continent. The gene pool of the fat-tailed sheep divided into the mountain type sheep (e.g. Andi and Lezgian) and steppe-desert types (e.g. Gala and Karakul). However, the majority of fat-tailed breeds have their ancestries in both of these subclusters (Figure 1), which together with low differentiation estimates indicates substantial gene flow between them. This agrees with the traditional sheep breeding practices in the Caucasus, which promote gene flow through the long-distance nomadic pasturing of animals. Grouping of Romanov sheep within the Fat-tailed cluster (Figure 1) should be regarded cautiously.

Decisions on adaptation conservation should largely be based on reliable phenotypic evaluations. In humans, genetic and phenotypic diversity agree [26], but selection might affect phenotypes reducing correlation between phenotypic divergence and general genomic relatedness [27,28]. This is particularly true for livestock which would imply need for testing (ecological) exchangeability (as in [29]). Unfortunately this is very difficult. A large proportion of the necessary phenotypic information exists only as informal knowledge of local breeders. Even the more rigidly collected data is rarely comparable between environments.

Molecular data can have a role in pointing out potential conservation gaps when phenotypic knowledge is limited. The usability of approaches based on molecular marker data in setting conservation priorities can be greatly improved by genome-wide surveys of molecular variation [30]. For example, scanning tens of thousands of SNP markers has the potential to identify selected loci [31] and allow comparison of the conservation values of several populations, both in the neutral and non-neutral context [30]. However, even with full genome sequences, valuation of populations can prove to be difficult due to incomplete understanding of the biology of the organisms and poorly definable conservation goals.

We used neutral molecular data for a specific set of populations and applied the method of Caballero and Toro [15] to calculate optimal contributions of Eurasian sheep breeds to the core set, which would minimize the mean kinship in the set and maximize N_e _and genetic diversity of the species. While giving more emphasis to divergence has theoretical appeal, it did not increase ecological or phenotypic heterogeneity in the preferred set of breeds compared with the maximization of global diversity (and N_e_). Maximization of global diversity prioritized a more diverse set of breeds originating from a range of biogeographic environments and having different genetic histories. Though the set looks reasonable, we acknowledge that it is based on incomplete data and we are hesitant to conclude that this particular design is optimal.

## Conclusions

Neutral variation suggested a general rule of thumb to favour breeds from the diversity hotspot regions in the first phase of *in situ *and *ex situ *conservation actions. In the final design, however, approximately equal population presentation across environments is recommended, but still higher per population emphasis in areas of high diversity is suggested. A comprehensive valuation of breeds, particularly within each physical environment, should consider production systems, important biological characteristics and available genetic information, as well as consideration of the probability of success and the extinction risk of breeds.

## Methods

### Biological samples

In total, 1675 animals representing 52 sheep breeds were studied (Additional file 2: Table S2). Sheep were sampled from three geographical regions: The Caucasus, Asia, and the eastern fringe of Europe, including central and western Russia. Each geographical region was further subdivided into regional groups. The Caucasian area was composed of the southern Caucasus (the following breeds were sampled: Azerbaijan Mountain Merino, Bozakh, Gala, Karabakh, Mazekh, Tushin), northern Caucasus (Andi, Dagestan local, Dagestan Mountain Merino, Karachai, Lezgian), Stavropol (Caucasian, North Caucasian Mutton-Wool, Stavropol), and the Caspian depression (Aksaraisk type of Soviet Mutton-Wool, Grozny, Volgograd). The Asian area was subdivided into the Kazakhstan and east of the Caspian Sea group (Degeres Mutton-Wool, Kazakh Arkhar-Merino, Kazakh Edilbai, Kazakh Finewool, Russian Edilbai, Russian Karakul), Altay (Gorno-Altay local, Kulunda), and the Buryatia group (Baidarak, Transbaikal Finewool). The remaining nine groups covered the eastern fringe of Europe: the Volga region (Kuibyshev, Oparin), western Russia (Kuchugur, Romanov, Russian Romney Marsh), Ukraine (Carpathian Mountain, Sokolsk), southeast Europe (Moldavial Karakul, Moldavial Tsigai, Pramenka, Russian Tsigai), Poland (Olkuska, Swiniarka, Wrzosowka), Finland (Finnsheep, Finnish Grey Landrace), Scandinavia (Swedish Rya Sheep, Swedish Gottland Sheep, Swedish Gute Sheep, Norwegian Rygja Sheep, Norwegian Cheviot, Norwegian Feral Sheep), Denmark (Danish Texel), and Iceland and the Faeroe Islands (Icelandic Sheep, Faeroe Island Sheep). Unrelated animals were sampled based on pedigree records (two previous generations) or farmers' knowledge.

Genomic DNA was extracted from blood as described in [32], or from skin samples using DNeasy Tissue Kit (Qiagen, Crawley, West Sussex, UK). Prior to DNA extraction, skin samples stored in ethanol were washed twice with phosphate buffered saline to remove fixatives.

### Genetic loci

The polymerase chain reactions (PCR) for 20 microsatellites (Additional file 1: Table S1) were performed as described in [33] and genotyped using the MegaBACE™ 500 DNA Sequencer (Amersham Biosciences). Fragment sizing was performed using the MegaBaceTM Genetic Profiler 2.2 or Fragment Profiler 1.2 (Amersham Biosciences). Genotypes for 20 microsatellites were available in the earlier studies for the Romanov sheep [34] and for the 11 breeds from Finland, Scandinavia, Denmark, Iceland and the Faeroe Islands [16].

### Statistical analysis

The microsatellite loci were characterized by the total number of alleles, expected heterozygosity or total gene diversity [35], sample-size-corrected allelic richness [36] corresponding here expected allele number in a sample of nine diploid individuals, and *F*-statistics using FSTAT v2.93 [37]. *F*-statistics were estimated using Weir and Cockerham [22] method where *f *and *θ *correspond to Wright's coefficients *F*_*IS *_and *F*_*ST*_, respectively. The genetic relationships among breeds were analyzed using principal coordinate analysis (PCoA) as implemented in PAST v1.73 [38] using the Chord distance [39].

A model-based Bayesian clustering analysis was used to infer population structure and the level of admixture in the sheep breeds implemented in STRUCTURE v2.2 [40]. The STRUCTURE algorithm assumes *K *populations, each of which is in Hardy-Weinberg and linkage equilibrium and characterized by a set of allele frequencies at each locus. Analysis was performed with a burn-in length of 20,000 followed by 100,000 Markov chain Monte Carlo iterations for each of *K *= 1 to 10, with ten replicate runs for each *K *using independent allele frequencies and an admixture model. Results across ten runs at each *K *were compared based on similarity coefficients (SC) as previously described in [41]. The breeds were assigned to wide clusters based on major ancestry and submitted to a second round of STRUCTURE analysis performed within each wide cluster.

A linear regression analysis was performed to study the influence of breed ancestry diversity (admixture) on the level of genetic diversity. Ancestry diversity for each breed was calculated as 1-Σ(q_k_) ^2^, where q_k _is an average fraction of the breed's genetic ancestry from the *k *separate genetic clusters at the optimal *K*, identified in STRUCTURE analysis. To examine the significance of mixed ancestries as sources of within-breed diversity, the obtained ancestry diversity values were compared with the unbiased expected heterozygozity estimates.

For the geographical plotting of genetic diversity parameters, latitude and longitude values for each breed were obtained from the center of the sample distribution. The ArcView GIS v9.1 (Environmental Systems Research Institute, ESRI, Redlands, CA, USA) was used to map the allelic richness and expected heterozygosity for each breed and the surface was extrapolated to a full rectangle. This was based on the Inverse Distance Weighted interpolation method [42], which assumes each input point to have a local influence that diminishes with distance. A synthetic map for the distribution of local total gene diversity (*H*_T_) and *θ *calculated for the geographically neighboring triplets of populations was done similarly. Population triplets were formed using Delaunay triangulation method implemented in the program Triangle [43].

Components of within- and between-breed genetic diversity were calculated based on the molecular coancestry for populations following the method described by Caballero and Toro [15]. The molecular coancestry between two individuals is the probability that two alleles at the locus taken at random from each individual are alike in state. In a structured population with *n *breeds the molecular coancestry between breeds *i *and *j *(*f*_*ij*_) is the average across loci and across individuals. Defining the within-breed average coancestry as $\tilde{f}=\frac{\sum_{i}f_{ii}}{n}$, the total population coancestry as $\bar{f}=\frac{\sum_{i,j}f_{ij}}{n^{2}}$, Nei's minimum distance as $D_{ij}=\frac{f_{ii}+f_{jj}}{\text{2}}-f_{ij}$ and the average Nei's minimum distance as $\bar{D}=\frac{\sum_{i,j}D_{ij}}{n^{2}}$, then the total gene diversity or expected heterozygosity $\left(GD_{T}=1-\bar{f}\right)$ is partitioned into components within breeds $\left(GD_{WS}=1-\tilde{f}\right)$ and another between breeds $\left(GD_{BS}=\tilde{f}-\bar{f}=\bar{D}\right)$.

The importance of different breeds has been calculated based on the contribution of each breed to a pool of animals or a core set that would maximize its genetic diversity (e.g. [15,44]). In the present study, the core set refers to the smallest set of sheep breeds that still encompasses the neutral genetic diversity in the species using the co-ancestry measure detailed above. These optimal contributions can also be applied with a weighted (λ) combination of within- and between-breed components of gene diversity $\lambda (1-\tilde{f})+\bar{D}$. Maximizing global diversity is achieved by giving equal weights to within- and between-breed diversity (λ = 1), while maximizing between-breed variation is achieved by ignoring within-breed diversity (λ = 0). Two intermediate λ values were recommended in earlier studies. Piyasatian and Kinghorn [45] suggested giving five times weight to the between breed variation as to the within-breed variation (λ = 0.2), reflecting the speed by which genetic change can be made across populations compared with selection within one large mixed population. Bennewitz and Meuwissen [46] proposed a weighting based on maximizing the total genetic variance of a hypothetical quantitative trait, which is equivalent by using a weighting factor of *λ *= 0.5. These four λ values were applied in estimating the optimal contributions using a simulated annealing algorithm [47].

## Authors' contributions

MT supervised the molecular analysis, consistency of allele calling, coordinated or performed statistical analysis and wrote the final drafts of the paper. MO did the genotyping and most of the writing and statistical analyses for the first draft. IT had significant contribution both to statistical analyses and manuscript writing. MAT contributed to analysis design and molecular co-ancestry based analyses. NM, MC, GG, TK and MM have collaborated in study design, sampling and interpretation of the results. In addition, MC and TK did part of the molecular analyses. JK was in charge of the overall study including it's design, sample collection, statistical analysis, manuscript writing and coordinating the author contributions. All authors read and approved the final manuscript.

## Supplementary Material

Additional file 1 **Table S1 - Marker diversity parameters**. PDF file with list of microsatellites and their chromosomal location, total number of alleles, expected unbiased heterozygosity, and estimates of within-population (*f*) and among-population (*θ*) fixation indices. Click here for file

Additional file 2 **Table S2 - Table of the name of sheep breeds, their origin, demographic status and diversity parameters**. PDF file with data on per population sample size, expected heterozygosity, within-breed fixation index (*f*), allelic richness, and number of private alleles. Click here for file

Additional file 3 **Figure S1 - Additional synthetic maps**. PDF file synthetic maps for within-breed diversity and breed differentiation. Click here for file

Additional file 4 **Table S3 - Breed-wise optimal contributions to a core-set for different weightings of the within-breed variation**. PDF file with detailed data summarized in Table 3. Click here for file

Additional file 5 **Table S4 - Distribution of core-set contributions using genetic clustering**. PDF file with table similar to Table 3, but using genetic clusters instead of regional groups to categorize breeds. Click here for file

Additional file 6 **Table S5 - Breeds, having equal contributions to the core set when the number of breeds conserved is fixed**. PDF file with table of included breeds when the number of included breeds is fixed at 5, 10, 15 or 20. Click here for file
