# Supplementary material for: Microsatellite-based genetic diversity and population structure of domestic sheep in northern Eurasia
Source: BMC Genet. 2010 Aug 10;11:76. doi: 10.1186/1471-2156-11-76 (PMC2931448; doi:10.1186/1471-2156-11-76)
Supplement: Additional file 1 — Table S1 - Marker diversity parameters. PDF file with list of microsatellites and their chromosomal location, total number of alleles, expected unbiased heterozygosity, and estimates of within-population (f) and among-population (θ) fixation indices. [file 1471-2156-11-76-S1.PDF]

Additional file 1: Table S1 - Marker diversity parameters

Microsatellites and their chromosomal location (Chr), total number of alleles (A_T), expected unbiased heterozygosity (H_E), and estimates of within-population (f) and among-population (θ) fixation indices.

Locus	Chr	A_T	H_E	f	θ
BM0757	9	14	0.739	-0.015	0.067*
BM1314	22	20	0.821	-0.019	0.080*
BM1818	20	19	0.835	0.003	0.070*
BM4621	6	24	0.848	0.019	0.085*
BM6506	1	12	0.621	-0.063	0.075*
BM6526	26	16	0.750	0.008	0.057*
BM8125	17	11	0.614	0.004	0.080*
CSSM31	23	25	0.809	0.027	0.061*
INRA23	1	16	0.834	0.013	0.058*
MAF36	22	19	0.783	-0.006	0.074*
MAF48	X	13	0.746	0.011	0.071*
MAF65	15	15	0.728	0.017	0.061*
McM527	5	14	0.759	0.031	0.074*
OarCP20	21	15	0.743	0.009	0.055*
OarCP34	3	10	0.728	0.003	0.084*
OarFCB128	2	19	0.770	0.033	0.068*
OarFCB304	19	34	0.724	0.026	0.090*
OarFCB48	17	18	0.729	0.005	0.059*
OarHH47	18	17	0.800	-0.008	0.059*
OarVH72	25	11	0.730	-0.028	0.079*
Mean		17	0.756	0.011	0.066

* Significantly different from zero at P<0.05, based on 10,000 permutations
