# Supplementary material for: Microsatellite-based genetic diversity and population structure of domestic sheep in northern Eurasia
Source: BMC Genet. 2010 Aug 10;11:76. doi: 10.1186/1471-2156-11-76 (PMC2931448; doi:10.1186/1471-2156-11-76)
Supplement: Additional file 2 — Table S2 - Table of the name of sheep breeds, their origin, demographic status and diversity parameters. PDF file with data on per population sample size, expected heterozygosity, within-breed fixation index (f), allelic richness, and number of private alleles. [file 1471-2156-11-76-S2.PDF]

Additional file 2: Table S2 – Table of the name of sheep breeds, their origin, demographic status and diversity parameters. Sample size (N), expected unbiased heterozygosity (H_E), within-breed fixation index (*f*), allelic richness (R), and number of private alleles (A_{PR}).

Breed	Country	Regional group	Latitude	Longitude	Demographic status	N	H_E	*f*	R	A_{PR}
Azerbaijan Mountain Merino	Azerbaijan	South Caucasus	40.6844N	46.3488E	Endangered	36	0.785	0.020	6.42	
Bozakh	Azerbaijan	South Caucasus	40.5287N	46.0230E	Rare	35	0.802	0.010	6.86	1
Gala	Azerbaijan	South Caucasus	40.5126N	47.6479E	Rare	31	0.794	0.026	6.56	1
Karabakh	Azerbaijan	South Caucasus	39.8223N	46.7011E	Rare	37	0.792	0.028	6.61	2
Mazekh	Azerbaijan	South Caucasus	40.5779N	46.3300E	Rare	33	0.772	0.020	6.28	
Tushin	Russia	South Caucasus	43.0318N	44.6742E	Endangered	39	0.799	0.016	6.69	
Andi	Russia	North Caucasus	42.7832N	46.4878E	Not at risk	38	0.764	0.027	5.88	1
Dagestan local	Russia	North Caucasus	42.7963N	46.5329E	Endangered	20	0.789	0.010	6.48	
Dagestan Mountain Merino	Russia	North Caucasus	43.6018N	46.7671E	Not at risk	39	0.761	0.027	5.84	
Karachai	Russia	North Caucasus	43.0440N	44.2095E	Not at risk	39	0.766	-0.001	6.38	
Lezgian	Russia	North Caucasus	43.1985N	46.8627E	Not at risk	40	0.799	0.022	6.62	1
Caucasian	Russia	Stavropol	45.7078N	42.8839E	Not at risk	37	0.791	0.007	5.97	
North Caucasian Mutton-Wool	Russia	Stavropol	44.7362N	43.3437E	Not at risk	37	0.775	-0.015	6.06	
Stavropol	Russia	Stavropol	46.3148N	44.2225E	Not at risk	24	0.783	-0.007	6.09	
Akasaraisk tp. of Soviet Mutton-Wool	Russia	Caspian depression	46.3699N	48.0945E	Not at risk	23	0.789	0.006	6.54	1
Grozny	Russia	Caspian depression	46.4969N	48.2664E	Not at risk	31	0.776	0.041	6.09	
Volgograd	Russia	Caspian depression	48.7103N	44.4836E	Not at risk	34	0.778	0.021	5.95	1
Degeres Mutton-Wool	Kazakhstan	Kazakhstan, east of Caspian Sea	43.2700N	76.8060E	Not at risk	40	0.767	-0.008	6.13	
Kazakh Arkhar-Merino	Kazakhstan	Kazakhstan, east of Caspian Sea	43.4528N	77.0350E	Not at risk	18	0.773	0.071	5.92	
Kazakh Edilbai	Kazakhstan	Kazakhstan, east of Caspian Sea	52.3236N	77.0305E	Not at risk	25	0.792	0.043	6.59	
Kazakh Finewool	Kazakhstan	Kazakhstan, east of Caspian Sea	42.9484N	71.4161E	Not at risk	25	0.789	0.046	6.34	
Russian Edilbai	Russia	Kazakhstan, east of Caspian Sea	45.2519N	45.8181E	Not at risk	32	0.794	0.021	6.75	3
Russian Karakul	Russia	Kazakhstan, east of Caspian Sea	45.3729N	46.0387E	Not at risk	37	0.806	0.050	6.98	4
Gorno-Altai local	Russia	Altai	49.9930N	88.6482E	Endangered	38	0.792	-0.009	6.41	1
Kulunda	Russia	Altai	51.9708N	82.9924E	Endangered	40	0.779	0.016	6.42	
Baidarak	Russia	Buryatia	50.8462N	105.4365E	Endangered	34	0.781	-0.020	6.42	
Transbaikal Finewool	Russia	Buryatia	50.9787N	105.7981E	Not at risk	35	0.795	0.005	6.42	
Kuibyshev	Russia	Volga region	52.8370N	49.0196E	Not at risk	38	0.786	0.039	6.21	
Oparin	Russia	Volga region	59.8489N	48.2840E	Extinct	22	0.747	-0.009	5.54	
Kuchugur	Russia	West Russia	51.6153N	39.4055E	Critical	40	0.713	-0.093	5.22	1
Romanov	Russia	West Russia	57.8516N	39.5259E	Not at risk	29	0.724	-0.008	5.12	
Russian Romney Marsh	Russia	West Russia	54.0011N	39.7789E	Endangered	33	0.754	-0.022	6.01	1
Carpathian Mountain	Ukraine	Ukraine	48.5511N	23.0096E	Not at risk	35	0.800	-0.004	6.42	1
Sokolsk	Ukraine	Ukraine	49.6308N	34.5851E	Not at risk	30	0.802	-0.004	6.44	3
Moldavian Karakul	Moldova	Southeast Europe	46.9336N	28.7462E	Not at risk	30	0.758	-0.008	6.07	
Moldavian Tsigai	Moldova	Southeast Europe	45.6886N	28.2969E	Not at risk	39	0.796	0.044	6.46	
Pramenka (Vlashko vitoroga)	Serbia	Southeast Europe	44.7362N	20.4367E	Critical	25	0.769	-0.026	5.75	
Russian Tsigai	Russia	Southeast Europe	51.4901N	46.0387E	Not at risk	33	0.791	-0.035	6.27	
Olkuska	Poland	Poland	49.7764N	21.3350E	Endangered	35	0.791	-0.004	6.57	
Swiniarka	Poland	Poland	50.0622N	19.9451E	Endangered	40	0.700	-0.040	4.87	1
Wrzosowka	Poland	Poland	49.6308N	20.2121E	Not at risk	40	0.751	-0.002	5.79	
Finnsheep	Finland	Finland	62.3067N	27.1709E	Not at risk	30	0.765	-0.009	5.77	1
Finnish Grey Landrace	Finland	Finland	66.9094N	26.1537E	Endangered	30	0.719	0.022	4.93	
Swedish Rya Sheep	Sweden	Scandinavia	62.0269N	14.4866E	Not at risk	31	0.667	0.079*	5.03	
Swedish Gotland Sheep	Sweden	Scandinavia	63.3567N	16.1698E	Not at risk	30	0.656	0.033	4.42	
Swedish Gute Sheep	Sweden	Scandinavia	57.5121N	18.4370E	Not at risk	20	0.637	0.034	4.08	2
Norwegian Rygja Sheep	Norway	Scandinavia	58.5874N	7.7960E	Not at risk	28	0.728	0.095*	4.97	1
Norwegian Cheviot Sheep	Norway	Scandinavia	61.6970N	9.8815E	Not at risk	28	0.613	-0.014	3.89	2
Norwegian Feral Sheep	Norway	Scandinavia	62.5337N	7.7496E	Rare	37	0.719	0.046	5.17	3
Danish Texel	Denmark	Denmark	56.1359N	9.4320E	Not at risk	24	0.651	0.028	4.41	
Icelandic Sheep	Iceland	Iceland and Faeroe Islands	64.4500N	19.3200W	Not at risk	30	0.741	0.053	5.37	1
Faeroe Island Sheep	Denmark	Iceland and Faeroe Islands	62.1353N	6.7687W	Not at risk	21	0.687	-0.056	4.54	

* Population estimate significantly larger ($P < 0.05$) than expected (after Bonferroni correction over populations) based on 20,800 randomizations of alleles within populations.
