# Supplementary material for: Microsatellite-based genetic diversity and population structure of domestic sheep in northern Eurasia
Source: BMC Genet. 2010 Aug 10;11:76. doi: 10.1186/1471-2156-11-76 (PMC2931448; doi:10.1186/1471-2156-11-76)
Supplement: Additional file 3 — Figure S1 - Additional synthetic maps. PDF file synthetic maps for within-breed diversity and breed differentiation. [file 1471-2156-11-76-S3.PDF]

Additional file 3: Figure S1 – Additional synthetic maps

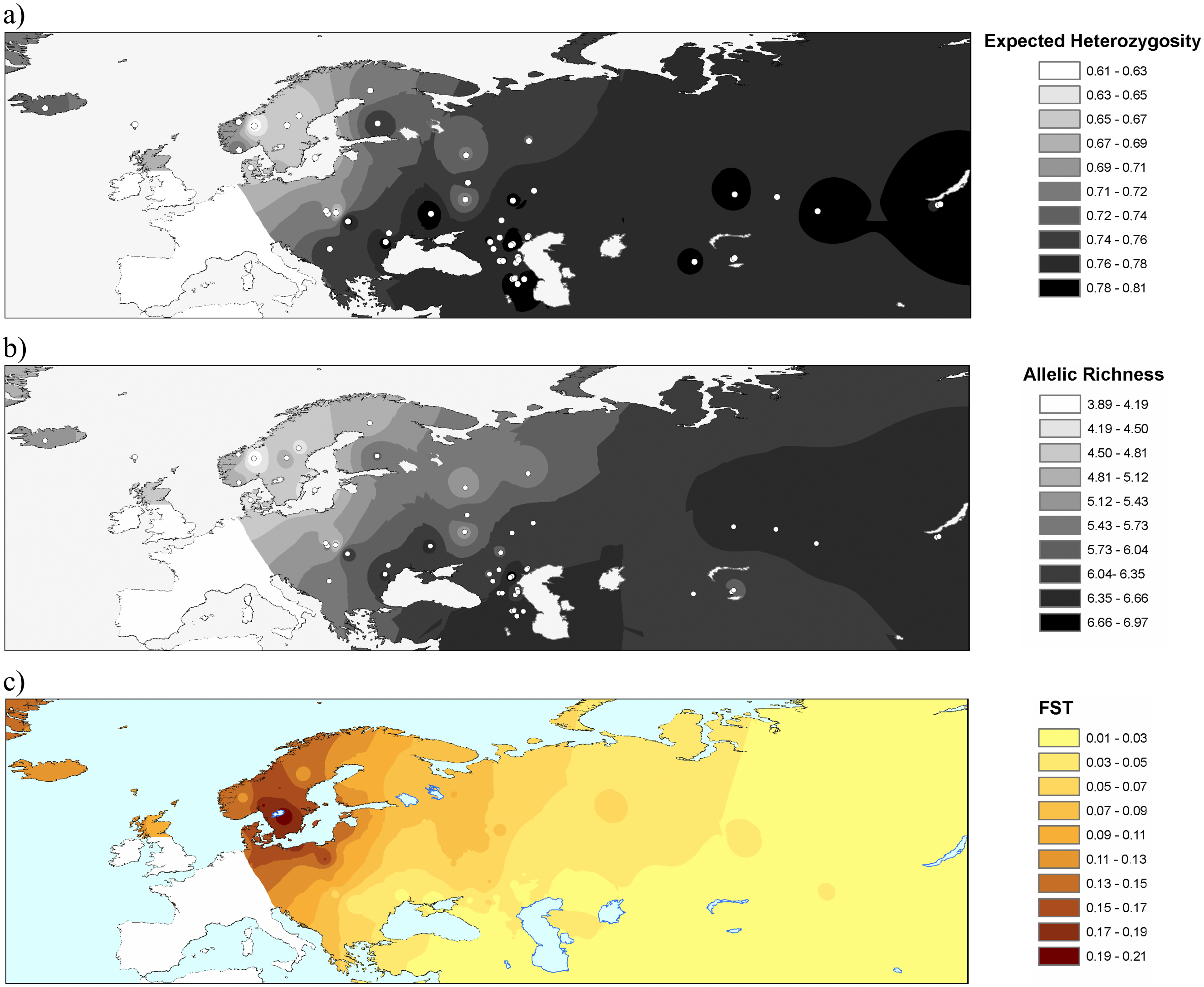

Contour synthetic maps of (a) expected heterozygosity and (b) allelic richness calculated for each breed. Darker shading indicates higher levels of variation. In (c) a contour synthetic map of θ calculated for each triplet set of breeds as described in the text. Light yellow shading indicates lower between breeds diversity.
