# Supplementary material for: Microsatellite-based genetic diversity and population structure of domestic sheep in northern Eurasia
Source: BMC Genet. 2010 Aug 10;11:76. doi: 10.1186/1471-2156-11-76 (PMC2931448; doi:10.1186/1471-2156-11-76)
Supplement: Additional file 4 — Table S3 - Breed-wise optimal contributions to a core-set for different weightings of the within-breed variation. PDF file with detailed data summarized in Table 3. [file 1471-2156-11-76-S4.PDF]

**Additional file 4: Table S3 – Breed-wise optimal contributions to a core-set for different weightings (λ) of the within-breed variation.**

| Geographical region | Regional group | Breed | λ=0 | λ=0.2 | λ=0.5 | λ=1 |
|---|---|---|---|---|---|---|
| Caucasus | South Caucasus | Azerbaijan Mountain Merino | 0 | 0 | 0 | 0 |
| | | Bozakh | 0 | 0 | 0 | 0 |
| | | Gala | 0 | 0 | 0 | 0.16 |
| | | Karabakh | 0 | 0 | 0 | 0.01 |
| | | Mazekh | 0 | 0 | 0 | 0 |
| | | Tushin | 0 | 0 | 0 | 0 |
| | North Caucasus | Andi | 0 | 0 | 0.04 | 0.01 |
| | | Dagestan local | 0 | 0 | 0 | 0 |
| | | Dagestan Mountain Merino | 0 | 0 | 0 | 0 |
| | | Karachai | 0 | 0 | 0 | 0 |
| | | Lezgian | 0 | 0 | 0 | 0 |
| | Stavropol | Caucasian | 0 | 0 | 0 | 0.03 |
| | | North Caucasian Mutton-Wool | 0 | 0 | 0 | 0 |
| | | Stavropol | 0 | 0 | 0 | 0.01 |
| | Caspian depression | Akasaraisk tp. of Soviet Mutton-Wool | 0 | 0 | 0 | 0 |
| | | Grozny | 0 | 0 | 0 | 0 |
| | | Volgograd | 0 | 0 | 0 | <0.01 |
| Asia | Kazakhstan and east of Caspian Sea | Degeres Mutton-Wool | 0 | 0 | 0 | 0 |
| | | Kazakh Arkhar-Merino | 0 | 0 | 0 | 0 |
| | | Kazakh Edilbai | 0 | 0 | 0 | 0 |
| | | Kazakh Finewool | 0 | 0 | 0 | 0 |
| | | Russian Edilbai | 0 | 0 | 0 | 0 |
| | | Russian Karakul | 0 | 0 | 0 | 0.14 |
| | Altay | Gorno-Altai local | 0 | 0 | 0 | 0 |
| | | Kulunda | 0 | 0 | 0 | 0 |
| | Buryatia | Baidarak | 0 | 0 | 0 | 0 |
| | | Transbaikal Finewool | 0 | 0 | 0 | 0 |
| Eastern fringe of Europe | Volga region | Kuibyshev | 0 | 0 | 0 | 0.06 |
| | | Oparin | 0 | 0 | 0 | 0 |
| | West Russia | Kuchugur | 0.04 | 0.08 | 0.10 | 0 |
| | | Romanov | 0 | 0.04 | 0.10 | 0.03 |
| | | Russian Romney Marsh | 0 | 0 | 0 | 0 |
| | Ukraine | Carpathian Mountain | 0 | 0 | 0 | 0.15 |
| | | Sokolsk | 0 | 0 | 0 | 0.09 |
| | South-east Europe | Moldavian Karakul | 0 | 0 | 0 | 0 |
| | | Moldavian Tsigai | 0 | 0 | 0 | 0 |
| | | Pramenka | 0 | 0 | 0.04 | 0.10 |
| | | Russian Tsigai | 0 | 0 | 0 | 0 |
| | Poland | Olkuska | 0.11 | 0.09 | 0.05 | 0 |
| | | Swiniarka | 0 | 0 | 0 | 0 |
| | | Wrzosowka | 0 | 0 | 0 | 0.02 |
| | Finland | Finnsheep | 0 | 0 | 0 | 0.03 |
| | | Finnish Grey Landrace | 0 | 0 | 0 | 0 |
| | Scandinavia | Swedish Rya Sheep | 0.10 | 0.10 | 0.08 | 0 |
| | | Swedish Gotland Sheep | 0.06 | 0.05 | 0.02 | 0 |
| | | Swedish Gute Sheep | 0.25 | 0.23 | 0.21 | 0.07 |
| | | Norwegian Rygja Sheep | 0 | 0 | 0.01 | 0.02 |
| | | Norwegian Cheviot Sheep | 0.16 | 0.13 | 0.08 | 0 |
| | | Norwegian Feral Sheep | 0 | 0 | 0 | 0 |
| | Denmark | Danish Texel | 0.19 | 0.17 | 0.13 | 0 |
| | Iceland and Faeroe Islands | Icelandic Sheep | 0 | 0 | 0.01 | 0.06 |
| | | Faeroe Island Sheep | 0.10 | 0.11 | 0.11 | 0 |
