# Supplementary material for: Microsatellite-based genetic diversity and population structure of domestic sheep in northern Eurasia
Source: BMC Genet. 2010 Aug 10;11:76. doi: 10.1186/1471-2156-11-76 (PMC2931448; doi:10.1186/1471-2156-11-76)
Supplement: Additional file 5 — Table S4 - Distribution of core-set contributions using genetic clustering. PDF file with table similar to Table 3, but using genetic clusters instead of regional groups to categorize breeds. [file 1471-2156-11-76-S5.PDF]

Additional file 5: Table S4 – Distribution of core-set contributions using genetic clustering

Number of breeds and the sum of optimal contributions (Cont) to the core set in each genetic cluster (based on STRUCTURE analysis) using four different weightings (λ) of the within-breed variation.

Geographical region	STRUCTURE cluster	Total no of breeds	λ=0		λ=0.2		λ=0.5		λ=1	
			Breeds	Cont	Breeds	Cont	Breeds	Cont	Breeds	Cont
Caucasus	Composite	8	0	0	0	0	0	0	3	0.04
	Fat-tailed	9	0	0	0	0	1	0.04	3	0.18
Asia	Composite	6	0	0	0	0	0	0	0	0
	Fat-tailed	4	0	0	0	0	0	0	1	0.14
Eastern fringe of Europe	Nordic	11	6	0.77	6	0.71	8	0.57	4	0.18
	Composite	12	2	0.23	2	0.25	3	0.27	5	0.42
	Fat-tailed	2	0	0	1	0.04	1	0.1	1	0.03
Total	Nordic		6	0.77	6	0.71	8	0.59	4	0.18
	Composite		2	0.23	2	0.25	3	0.27	8	0.46
	Fat-tailed		0	0	1	0.04	2	0.14	5	0.36
