# Supplementary material for: Microsatellite-based genetic diversity and population structure of domestic sheep in northern Eurasia
Source: BMC Genet. 2010 Aug 10;11:76. doi: 10.1186/1471-2156-11-76 (PMC2931448; doi:10.1186/1471-2156-11-76)
Supplement: Additional file 6 — Table S5 - Breeds, having equal contributions to the core set when the number of breeds conserved is fixed. PDF file with table of included breeds when the number of included breeds is fixed at 5, 10, 15 or 20. [file 1471-2156-11-76-S6.PDF]

Additional file 6: Table S5 – Breeds, having equal contributions to the core set when the number of breeds conserved is fixed.

Geographical region	STRUCTURE cluster	Total no of breeds	5 breeds	10 breeds	15 breeds	20 breeds
Caucasus	Composite	8	-	-	Caucasian	Caucasian<br>Volgograd<br>Stavropol
	Fat-tailed	9	Gala*	Gala*<br>Karabakh*	Gala*<br>Karabakh*<br>Bozakh*<br>Andi	Gala*<br>Karabakh*<br>Bozakh*<br>Andi<br>Tushin*<br>Lezgian*
Asia	Composite	6	-	-	-	-
	Fat-tailed	4	Russian Karakul*	Russian Karakul*	Russian Karakul*	Russian Karakul*
Eastern fringe of Europe	Nordic	11	-	Swedish Gute Sheep<br>Icelandic Sheep	Swedish Gute Sheep<br>Icelandic Sheep<br>Norwegian Rygja	Swedish Gute Sheep<br>Icelandic Sheep<br>Norwegian Rygja<br>Finnsheep
	Composite	12	Carpathian Mountain*<br>Kuibyshev<br>Pramenka*	Carpathian Mountain*<br>Kuibyshev<br>Pramenka*<br>Sokolsk*	Carpathian Mountain*<br>Kuibyshev<br>Pramenka*<br>Sokolsk*<br>Wrzosowka	Carpathian Mountain*<br>Kuibyshev<br>Pramenka*<br>Sokolsk*<br>Wrzosowka
	Fat-tailed	2	-	Romanov	Romanov	Romanov

* Breeds from diversity hotspot areas.
